# Bacteria-derived chimeric toxins as potential anticancer agents

**DOI:** 10.3389/fonc.2022.953678

**Published:** 2022-09-07

**Authors:** Saeed Khoshnood, Hadis Fathizadeh, Foroogh Neamati, Babak Negahdari, Piyush Baindara, Mohd Azmuddin Abdullah, Mohammad Hossein Haddadi

**Affiliations:** ^1^ Clinical Microbiology Research Centre, Ilam University of Medical Sciences, Ilam, Iran; ^2^ Student Research Committee, Sirjan School of Medical Sciences, Sirjan, Iran; ^3^ Department of Laboratory Sciences, Sirjan School of Medical Sciences, Sirjan, Iran; ^4^ Department of Microbiology, Faculty of Medicine, Kashan University of Medical Sciences, Kashan, Iran; ^5^ Department of Medical Biotechnology, School of Advanced Technologies in Medicine, Tehran University of Medical Sciences, Tehran, Iran; ^6^ Department of Molecular Microbiology and Immunology, School of Medicine, University of Missouri, Columbia, MO, United States; ^7^ Department of Toxicology, Advanced Medical and Dental Institute, Universiti Sains Malaysia, Bertam Campus, Kepala Batas, Pulau Pinang, Malaysia

**Keywords:** anticancer, chimeric toxin, bacterial toxins, immunotoxin, affibody, bacteria-derived chimeric toxin, exotoxin A, ligand-based immunotoxins

## Abstract

Cancer is one of the major causes of death globally, requiring everlasting efforts to develop novel, specific, effective, and safe treatment strategies. Despite advances in recent years, chemotherapy, as the primary treatment for cancer, still faces limitations such as the lack of specificity, drug resistance, and treatment failure. Bacterial toxins have great potential to be used as anticancer agents and can boost the effectiveness of cancer chemotherapeutics. Bacterial toxins exert anticancer effects by affecting the cell cycle and apoptotic pathways and regulating tumorigenesis. Chimeric toxins, which are recombinant derivatives of bacterial toxins, have been developed to address the low specificity of their conventional peers. Through their targeting moieties, chimeric toxins can specifically and effectively detect and kill cancer cells. This review takes a comprehensive look at the anticancer properties of bacteria-derived toxins and discusses their potential applications as therapeutic options for integrative cancer treatment.

## 1 Introduction

Cancer is the uncontrolled growth of cells leading to the formation of tumors that can metastasize to various body organs (1, 2). Despite recent therapeutic advances, cancer remains one of the major causes of death worldwide due to profound therapeutic challenges (2). According to the GLOBOCAN report, 19.3 million new cancer cases and 10 million cancer-related deaths have been registered in the world in 2020 (3). The wide application of chemotherapy is still facing problems associated with nonspecific targeting, lack of specificity, drug resistance, and disease recurrence (4). Bacteria can be helpful in treating cancer *via* producing various cytotoxic agents, toxins, and prodrug-modifying enzymes (5). Bacteria-derived toxins and antimicrobial peptides (AMPs) have great potential as novel, effective, specific, and safe anticancer agents (6).

**Figure 1:**
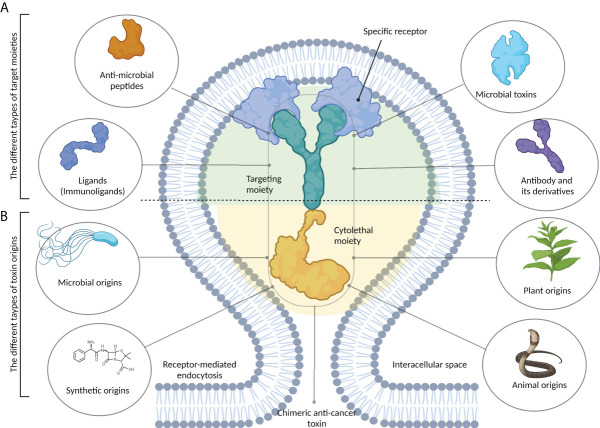
The structure of chimeric anticancer toxins (CATs). These chimeric toxins contain two distinct components. **(A)** A target moiety that is responsible for recognizing cancer-specific receptors on tumor cells. This part can be derived from different types of biomolecules, including antibodies and their derivatives, microbial toxins, antimicrobial peptides, and immunoligands. **(B)** A cytolethal moiety that is responsible for killing the host cell. Chimeric toxins can be developed from the primary toxins that may be synthetic or obtained from different sources, including microbes, plants, and animals.

Toxins and their recombinant derivatives possess potent anticancer properties and can specifically and selectively target tumor cells (7). As seen in **Figure 1**, chimeric anticancer toxins (CATs) consist of two distinct subunits: a targeting moiety and a cytolethal moiety. The presence of a targeting moiety, including a recognition moiety derived from bacterial toxins, monoclonal antibodies, immunoligands, and anionic or cationic AMPs, allows CATs to specifically target cancer cells *via* binding to their surface receptors (4).

Immunotoxins, which are versatile bacteria-derived CATs approved by the US Food and Drug Administration (FDA) for clinical use, have been studied as anticancer agents (8–10). A list of FDA-approved bacteria-derived immunotoxins has been given in **Table 1**.

**Table 1:** The list of FDA-approved bacterial-derived immunotoxins with anti-cancer properties.

Generic name	Brand name	Health resources	Toxin moiety	Targetedmoiety	Molecular weight	Expression system	Ref
Denileukin diftitox	Ontak®	Ontak® is indicated for treating adult patients with refractory or recurrent CTCL whose malignant cells express CD25 (a component of IL-2 receptor).	rDT	Il-2	58 kDa	*E. coli*	(1)
Tagraxofusp-erzs	Elzonris®	Elzonris® is a CD123-directed cytotoxin used for treating BPDCN in adults and children of 2 years of age or older.	tDT	IL-3	57 kDa	*E. coli*	(2)
Moxetumomab pasudotox-tdfk	Lumoxiti®	Lumoxiti® is indicated for treating adult patients with relapsed or refractory HCL, who have received at least two prior systemic therapies, including treatment with PNA.	tPE	CD22	63 kDa	*E. coli*	(3)

1. Foss F, editor Clinical experience with denileukin diftitox (ONTAK). Seminars in oncology; 2006: Elsevier.

2. Syed YY. Tagraxofusp: first global approval. Drugs. 2019;79(5):579-83.

3. Dhillon S. Moxetumomab pasudotox: first global approval. Drugs. 2018;78(16):1763-7.CTCL, Cutaneous T-cell lymphoma; IL-2, Interleukin-2; rDT, recombinant diphtheria toxin; *E. coli, Escherichia coli*; tPE, truncated Pseudomonas exotoxin.

There is no comprehensive review on bacteria-derived CATs, including immunotoxins and ligand-, AMP-, and affibody-based CATs. We also elaborated the concept of CATs using bacteria-derived proteins (i.e., toxins, AMPs, and affibodies) for cancer therapy and reviewed the anticancer properties, key features, and preparation protocols of bacteria-derived CATs.

## 2 Bacterial toxins for fighting against cancer

**Figure 2:**
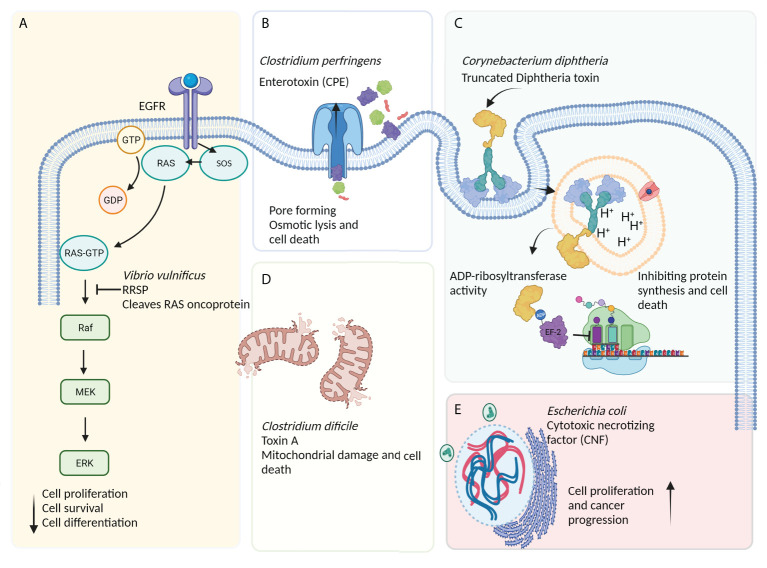
The effects of bacteria-derived toxins on cancerous cells. **(A)** Ras/Rap1-specific endopeptidase (RRSP) toxin secreted by *Vibrio vulnificus* blocks the RAS signal transduction pathway, leading to the abrogation of key signaling modulators (especially Raf) and a reduction in cell proliferation, differentiation, and, ultimately, survival. **(B)** Apoptosis induced by excessive osmotic pressure caused by the action of pore-forming toxins such as *Clostridium perfringens* enterotoxin (CPE) and *Aeromonas hydrophila* aerolysin. **(C)** The receptor-mediated internalization of diphtheria toxin (DT)-based immunotoxin blocks protein synthesis *via* inducing the ADP-ribosylation of elongation factor-2 (EF-2), leading to ADP-ribosyl transferase-mediated apoptosis. **(D)** Other bacterial toxins, such as toxin A (produced by *Clostridium difficile*), can induce mitochondria damage and, subsequently, cell death. **(E)** The cytotoxic necrotizing factor (CNF) is a bacterial single-chain exotoxin produced by Gram-negative bacteria, such as *Escherichia coli*, and promotes oncogenesis through inducing the activation and proliferation of host cells *via* a Rho-GTPase-dependent mechanism.

In recent years, bacteria-assisted immunotherapy has been proven to be a promising approach for combating cancer. During bacteriolytic tumor therapy (BTT), bacteria produce cytotoxic proteins, such as toxins and immune-modulating factors, that inhibit tumor growth and cell proliferation (11). Bacteria produce a variety of toxins, some of which can kill cancer cells by inducing different apoptotic pathways, regulating tumorigenesis processes (e.g., proliferation, differentiation, and apoptosis), and suppressing cancer progression (**Figure 2**) (12). A number of bacterial toxins can affect cell cycle progression. For example, *Escherichia coli* produces a toxin called cytotoxic necrotizing factor (CNF) that induces DNA replication, leading to the formation of multinucleated cells secondary to the suppression of cell differentiation and apoptosis induction (5). Exotoxin A secreted by *Pseudomonas aeruginosa* affects mRNA translation, promoting cytotoxicity against cancer cells (13). This toxin has been shown to inactivate poly ADP-ribose polymerase (PARP), induce caspase 3-dependent apoptosis and DNA fragmentation, impair endoplasmic reticulum function, and increase intracellular calcium levels in melanoma cells (14). In a recent study on animal models and human and murine cancer cell lines, it was suggested that *P. aeruginosa* exotoxin T (ExoT) could play a role as a potential anticancer agent (15). Human gut is often a reservoir of *Klebsiella pneumonia*, a microbe that contributes to colorectal cancer development. It was recently found that colibactin produced by *pks*-positive *K. pneumonia* could cause inflammation and DNA damage during the progression of colorectal cancer (16). Other studies have shown that *Clostridium perfringens* enterotoxin (CPE), a pore-forming bacterial toxin, can induce necrosis in tumors, inhibit the growth of cancer cells, and prevent tumor development (17, 18). *Corynebacterium diphtheria* secretes a toxin called diphtheria toxin (DT), which, like *Pseudomonas* exotoxin (PE), inhibits protein synthesis, offering a promising anticancer candidate (19). Ansiaux and Gallez (20) evaluated the cytotoxic effects of *Clostridium botulinum* neurotoxin (BoNT) and showed the vasodilatory effects of this toxin on tumor vessels, boosting the effectiveness of chemotherapy and radiotherapy. The lack of specificity of some bacterial toxins has led to the production of CATs [i.e., toxins conjugated with monoclonal antibodies (scFv and nanobody), other anticancer compounds, antibodies, peptides, or enzymes] *via* genetic engineering techniques to increase the efficiency of toxins (21).

## 3 Chimeric anticancer toxins with bacteria-derived moieties

**Figure 3:**
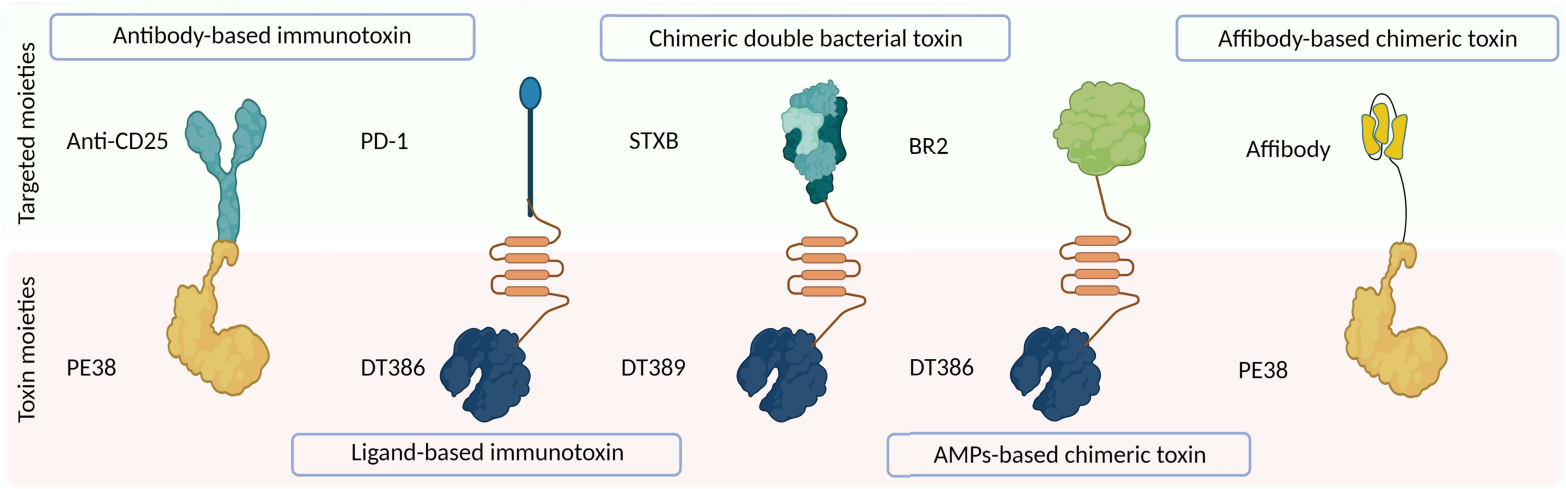
The schematic representation of different types of bacteria-derived chimeric toxins. DT, diphtheria toxin; truncated DTs, DT386 and DT389; STXB, Shiga-like toxin-B; BR2, buforin II; PE, *Pseudomonas* exotoxin A; PD1, programmed cell death protein-1.

Off-target toxicity is a major obstacle reducing the efficiency of bacterial toxins during cancer therapy. Designing CATs can be considered a foremost approach to generate novel toxins with low off-target toxicity and desirable immunogenicity (22). CATs contain two functional moieties: 1) targeting portion and 2) cytolethal part. The targeting moiety enables toxins to directly interact with their targets. Based on their origin, targeting moieties are divided into three categories: 1) antibody-derived, ligand-based, and bacteria-derived toxin derivatives; 2) AMPs; and 3) affibodies (**Figure 1**). **Figure 3** illustrates five main types of bacteria-derived CATs.

The cytolethal moiety alters the function of a variety of proteins and disrupts cellular signaling pathways, leading to the direct or indirect killing of intoxicated cells. This moiety can be derived from different origins such as bacteria, fungi, plants, animals, and synthetic drugs (**Figure 1**) (23). In the following section, we reviewed CATs containing at least one bacteria-derived moiety (**Figure 3**).

### 3.1 Immunotoxins

The high specificity of monoclonal antibodies renders them highly efficient and specific tools for targeting purposes, increasing their penetration into and retention in tumors and improving the antitumor efficacy of cancer immunotherapy (24). The efficacy of monotherapy by monoclonal antibodies is limited due to problems such as therapy resistance and disease relapse, requiring the development of new generations of monoclonal antibody-based anticancer drugs such as immunotoxins (25).

Immunotoxin is a protein consisting of a cytotoxic component and a targeting component, typically an antibody or its derivative, conferring specific elimination of targets. For immunotoxins to be effective, the toxin moiety must be internalized into the cytosol so that it can inhibit cellular protein synthesis. The cytosol delivery of the toxin is mediated by the targeting moiety (26).

**Table 2** summarizes clinical trials on the effectiveness of immunotoxin in cancer therapy. Although many bacteria can produce a variety of toxins, only two can be regarded as salient, *C. diphtheria*-derived DT and *P. aeruginosa*-derived PE (27). There are, however, many bacterial toxins that can be used to produce immunotoxins. In the following section, we introduced immunotoxins containing various bacteria-derived cytolethal moieties.

**Table 2:** The list of clinical trials of immunotoxin therapy in cancer.

Toxin moiety	Targeted moiety	Diseases and conditions	Intervention	Status (Phase)	NCT
PE	Anti-mucin 1 (BM7)	Colorectal Cancer Metastatic	BM7PE	Recruiting (I & II)	04550897
	MOC31	Colorectal Neoplasms	MOC31PE	Completed (I & II)	02219893
PE38	Anti-Tac murine (Anti CD-25)	Leukemia, Lymphoma	LMB-2	Completed (I)	00002765
					00085150
		CTCL			00080535
		CLL			00077922
		HCL		Active, not recruiting (I)	00321555
		Melanoma (Skin) Non-melanomatous Skin Cancer	LMB-2 , MART-1 antigen, gp100 antigen, In-Freund's adjuvant	Completed (I)	00295958
		ATL	LMB-2, Fludarabine, Cyclophosphamide	Active, not recruiting	00924170
	SS1(dsFv), Anti-Mesothelin	Advanced cancers, Mesothelin expressing cells	SS1(dsFv)-PE38	Completed (I)	00006981
					00066651
		Non-Small Cell Lung Cancer Adenocarcinoma	SS1 (dsFv) PE38, Paclitaxel, Carboplatin, Bevacizumab		01051934
		Mesothelioma	Multicycle SS1P, Pemetrexed, Cisplatin, Single cycle SS1P	Terminated (I)	01445392
		Mesothelioma, Adenocarcinoma of Lung, Pancreatic Neoplasms	Pentostatin, Cyclophosphamide, SS1(dsFv)PE38	Completed (I & II)	01362790
	Anti-MOC31	Carcinoma	MOC31PE	Complete (I)	01061645
		Colorectal Neoplasms		Completed (I & II)	02219893
	anti-Lewis Y. (B3 scFv)	Brain and CNS Tumors	LMB-7	Complete (I)	00003020
	disulfide-stabilized Fv (dsFv) of B3	Bladder Cancer, Breast Cancer, Colorectal Cancer, Lung Cancer Ovarian Cancer, Pancreatic Cancer	LMB-9	Completed (I)	00005858
		Advanced solid tumors including: Bladder Cancer, Breast Cancer, Colorectal Cancer, Esophageal Cancer, Gastric Cancer, Lung Cancer, Pancreatic Cancer			00019435
		Colorectal Cancer, Esophageal Cancer, Gastric Cancer, Pancreatic Cancer		Unknown (I)	00010270
	Anti-Tac murine (Anti CD-25)	ATL	LMB-2, Fludarabine, Cyclophosphamide	Active, not recruiting	00924170
	Anti-CD22 FV (RFB4)	Leukemia	BL22	Completed (I)	00021983
					00074048
		Leukemia, Lymphoma			00126646
	Modified anti-CD22 FV (RFB4)	Leukemia	CAT-8015	Unknown (I)	00457860
		Leukemia, HCL			00462189
		HCL	CAT-3888	Terminated (I)	00924040
	Anti-CD22 FV	HCL	Lumoxiti®	Recruiting (I)	03805932
		ALL		Terminated (I)	02338050
		R/R HCL		Approved for marketing	03501615
		HCL	Lumoxiti®, IV Bag	Completed (I &II)	01829711
PE38QQR	Anti-IL-13	BCNST	Cintredekin besudotox	Completed (I)	00036972
			Cintredekin besudotox isolated perfusion	Completed (I & II)	00006268
		malignant brain tumors	Cintredekin besudotox	Completed (I)	00064779
PE38KDEL	Anti-EGFRvIII (MR1scFv)	Supratentorial Malignant Brain Tumor	MR1-1	Terminated (I)	01009866
	Anti-IL-4	BCNST	IL-4(38-37)-PE38KDEL	Unknown (I)	00003842
PE24	Human anti-mesothelin (Fab)	Neoplasms With Mesothelin	LMB-100, Tofacitinib	Active, not recruiting (I)	04034238
		Neoplasms, Pancreatic Neoplasms	LMB-100, Nab-Paclitaxel	Complete (I & II)	02810418
		Mesothelioma	LMB-100, Nab-Paclitaxel	Complete (I)	02798536
		Cancers Expressing Mesothelin,	LMB-100	Not yet recruiting (I)	05375825
	Anti-mesothelin Fab	Mesothelioma	LMB-100, Pembrolizumab	Terminated (I)	03644550
			LMB-100, SEL-110		03436732
			LMB-100, Ipilimumab	Recruiting (I)	04840615
DT390	Anti-CD19/CD22 bispecific	R/R B-Lineage Leukemia and lymphoma	DT2219ARL	Completed (I & II)	02370160
		Leukemia, Lymphoma		Completed (I)	00889408
rDT	Anti-transferin	Brain and CNS Tumors	transferrin-CRM107	Unknown,	00052624
DT389	Anti-CD3	ATLL, SS, MF, CTCL	UCHT1	Completed (I)	00611208
	Anti-IL-2	Leukemia, Adult T-Cell	Denileukin diftitox (Ontak)	Terminated (I)	00117845
SLTA	anti-CD20	NHL, Lymphocytic, Chronic, SLL, DLBCL, Blood Cancer, Hematological Malignancy	MT-3724	Terminated (I & II	02361346

ATLL, Adult T-cell leukaemia/lymphoma; CLL, Chronic lymphocytic leukemia; CNS, Central Nervous System; SS, Sezary Syndrome; CTCL, Cutaneous T-cell lymphoma; DLBCL, Diffuse large B cell lymphoma; DT, Diphtheria Toxin; R/R, Refractory /relapsed; SLTA, Shiga-like toxin A; HCL, Hairy cell leukemia; MF, Mycosis Fungoides; NHL, Non-Hodgkin lymphoma; PE, Pseudomonas exotoxin; SLL, Small lymphocytic lymphoma.

#### 3.1.1 *Pseudomonas aeruginosa* exotoxin a-based immunotoxins

The inhibition of protein synthesis is a common pathway for toxin-related apoptosis, which is triggered by the ADP-ribosylation of elongation factor 2 (eEF-2) (28). PE belongs to the mono-ADP-ribosyl transferase family, facilitating the production of ribosylated-eEF-2 and the disruption of protein synthesis. In this manner, the delivery of specific bacterial toxins to tumor cells *via* antibody-specific targeting can induce ADP-ribosylation-dependent killing (13).

Exotoxin A is synthesized as an inactive protein. Extracellular PE toxin consists of 613 amino acid residues and is trimmed at the N-terminal domain before being secreted. Furin-protease activity is essential for the endosomal activation of the toxin and plays a critical role in its cytotoxicity and translocation *via* a retrograde trafficking route (29). The native PE consists of different functional domains that have been manipulated to develop novel chimeric toxins. An example of these is moxetumomab pasudotox (Lumoxiti^®^), an immunotoxin consisting of anti-CD22-dsFV and PE38 (**Table 1**) (30).

**Figure 4:**
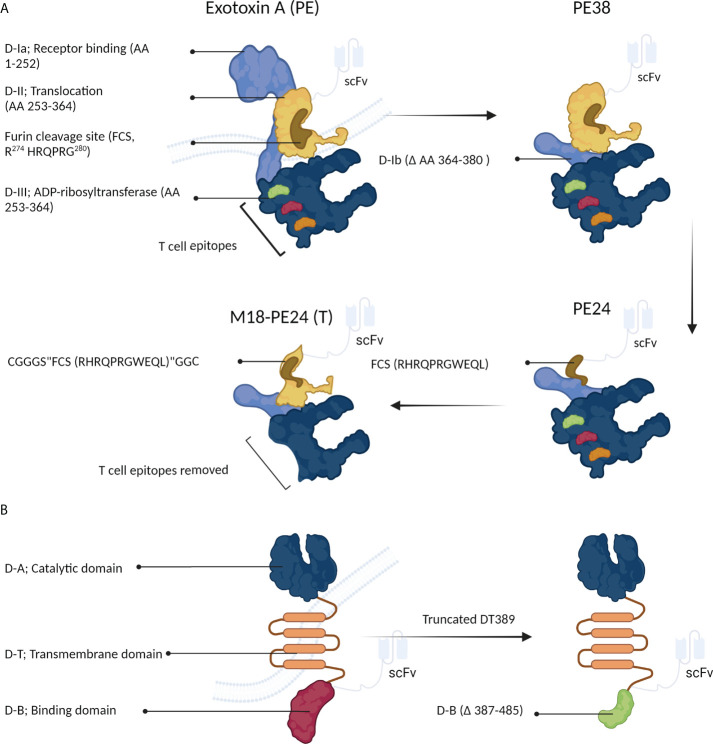
The structure of *Pseudomonas* exotoxin A (PE) and diphtheria toxin (DT). **(A)** PE can be manipulated to develop immunotoxins. **(B)** Structural changes in DT can increase its anticancer activity.

**Figure 4A** schematically shows the optimization process of PE-derived CATs. Exotoxin A is composed of three domains; 1) D-I (which is divided into the subdomains of D-Ia, i.e., a part that can be removed during engineering and can directly recognize and bind CD19 on eukaryotic cells, and D-Ib, i.e., a structural subunit), 2) D-II, and 3) D-III. In acidic endocytic vesicles, the D-II domain is hydrolyzed by a furin-like protease at the arginine-279 residue. This proteolytic cleavage creates a new PE toxin containing the D-I and D-III domains and is essential for assembling the active toxin (29). Next, the D-I/D-III complex arrives at the endoplasmic reticulum from the Golgi apparatus, where D-III is released by protein disulfide isomerases and translocated into the cytosol *via* the Sec61p translocon. During the release, the D-I domain is lost, and only the D-III domain (which possesses ADP-ribosyl transferase activity) remains (13).

For the first time, Siegall et al. (31) designed a recombinant PE, known as PE38, which lacked amino acids 360-380 of the D-Ib domain, making PE38 shorter than the native PE. The D-III domain along with a part of the D-II domain is crucial for the ADP-ribosyl transferase activity of PE. Various recombinant PE toxins, such as PE40, PE38, PE38QQR, PE38KDEL, PE24, and M18-PE24, have been proposed and used in the clinical setting (**Table 2**) (13, 32–35).

The anticancer efficacy of moxetumomab pasudotox immunotoxin (Lumoxiti^®^) has been investigated in four clinical trials (**Table 2**). A phase II clinical trial had been conducted on patients with relapsed/refractory hairy cell leukemia (R/R HCL) for marketing approval. In another study (NCT03501615), 16.7 months of moxetumomab pasudotox therapy led to a durable complete response in 30% of patients, while 75% showed an objective response. A delayed complete response appeared in five of the patients after 6 months of therapy initiation (NCT02912754). Moxetumomab pasudotox immunotoxin is the only PE-based immunotoxin approved by the FDA (**Table 1**) (36).

The therapeutic efficiency of PE-derived immunotoxins in clinical studies has been limited due to undesirable features of PE in terms of immunogenicity, targeted toxicity, off-target systemic toxicity, and the development of therapy resistance (37–39). Anti-Tac(Fv)-PE38humanized antiTac toxin (LMB-2) is another immunotoxin consisting of scFv anti-CD25 and PE38, showing high cytotoxicity against hematological malignant cells in adult T-cell leukemia, B-cell chronic lymphocytic leukemia, anaplastic large-cell lymphoma, B-cell non-Hodgkin lymphoma, Hodgkin’s disease, and HCL. Nevertheless, 37% of LMB-2 recipients either developed anti-LMB-2 antibodies or showed off-target toxicity and side effects such as the vascular leak syndrome (VLS) (35).

Recently, to minimize toxin immunogenicity and off-target toxicity, Kaplan et al. (35) fabricated a recombinant PE38 to develop new CD22-targeting immunotoxins. In this regard, PE38 was genetically modified by introducing six point mutations into the D-III domain and removing the D-II domain, except for the furin cleavage site (FCS; “RHRQPRGWEQL”), making a PE with low immunogenicity, known as PE24. This novel toxin was shown to reduce LMB-2 T cell-mediated immunogenicity (**Figure 4A**). Although the lack of the D-II domain in the newly generated PE (PE24) immunotoxin reduced its ability to kill HUT-102 cells compared to the parental immunotoxin (i.e., PE38) (IC_50s_ of 3.4 and 0.1 µM for PE24 and PE38, respectively), the cytotoxic activity was largely recovered by constructing the chimeric M18-PE24 toxin (IC_50_ = 0.7 µM, **Figure 4A**). The removal of the T-cell epitope is a technique approved for reducing the immunogenicity of therapeutic proteins. The T-cell epitopes of D-III PE, including R427A, F443A, L477H, R494A, R505A, and L552E, are responsible for its high immunogenicity. The M18-PE24 (T) recombinant toxin showed low immunogenicity and high cytotoxic activity compared to its parental toxin (i.e., M18-PE24) (35). Overall, the manipulation of the D-II and D-III domains seems to be a promising way to generate more effective PE-based chimeric toxins for fighting against cancer (**Figure 4A**).

Human epidermal growth factor receptor 2 (HER2) is overexpressed in many tumors such as breast and gastric, suggesting this receptor as an attractive therapeutic target to obviate problems caused by low immunogenicity and off-target toxicity. Recently, due to resistance to conventional drugs (such as trastuzumab) in breast and gastric tumors, efforts have been directed to develop new PE-based immunotoxins (IHP25-BT) (40). Guo et al. (40, 41) developed a new series of HER2-targeting PE25-based immunotoxins that showed significant *in vitro* antitumor activity against two trastuzumab-resistant cell lines (NCI-N87-TR and BT474-TR). These PE25-based immunotoxins delivered high efficacy, low immunogenicity, and negligible off-target toxicity compared to PE38-based immunotoxins. Particularly, IHP25-BT delivered a desirable maximum tolerated dose (MTD) in mice and potent antiproliferative activity *in vitro*, rendered great antitumor potency against NCI-N87 cells in NCI-N87-TR nude xenograft mice, and, finally, reduced liver metastasis. These findings suggested that removing B- and T-cell epitopes from the D-III domain and replacing the D-II segment (△251–273 and △285–394) of PE38 with a furin-cleavable linker could significantly enhance the antiproliferative and antimetastasis activities of the PE25 chimeric toxin (**Figure 4A**) (40).

Mesothelin (MSLN) is a tumor differentiation antigen normally expressed on the mesothelial cells lining the pleura, peritoneum, and pericardium. Weldon et al. (42) showed that a PE25-conjugated anti-MSLN antibody delivered high anticancer activity in mice and low antigenicity and low precipitation by anti-SS1P (parental PE38) in human sera. RG7787 is a recombinant derivative of PE carrying point mutations (alanine) in seven bulky hydrophilic residues of PE24, enhancing its B-cell epitope-related immunogenicity (43). RG7787-conjugated MSLN is being evaluated in phase I clinical trials on patients with MSLN-positive malignancies, including mesothelioma and ovarian, pancreatic, gastric, and triple-negative breast cancer (TNBC) (**Table 2**) (44). In addition to MSLN, paclitaxel-conjugated RG7787 has been recently shown to successfully induce remission in mouse models of pancreatic cancer (9).

While the use of immunotoxin-based therapy is expanding to a broad range of cancers, the efficiency of this method has been compromised by the emergence of immunotoxin resistance, which has been comprehensively reviewed by Dieffenbach and Pastan (38). Altogether, low immunogenicity, off-target toxicity, and drug resistance necessitate the development of more effective PE38-based immunotoxins in the future.

#### 3.1.2 *Corynebacterium diphtheria* toxin-based immunotoxins

The development of a chimeric toxin efficient in killing cancer cells is a major concern. Because of its high toxicity against cancerous cells, high expression, and minimal side effects, DT has been recently used to develop anticancer immunotoxins (45). The DT toxin consists of three distinct domains, binding (B), catalytic (A), and transmembrane (T) domains, and is a well-recognized cytotoxic protein that mediates direct cytolethal effects against target cells (**Figure 4B**) (45). This toxin rapidly suppresses the protein synthesis system. The “B” and “T” domains are responsible for the toxin’s specific binding and cytoplasmic translocation, respectively. Once released into the cytoplasm of host cells, the “A” domain mediates the transference of an adenosine diphosphate ribosyl (ADPR) moiety onto the EF-2 factor (**Figure 2C**) (46). DT-based immunotoxins have exhibited potent *in vitro* antitumor activity (IC_50_s =10^-9^–10^-14^), suggesting it as a suitable agent to be used in targeted cancer therapy (45).

Different truncated forms of DT have been used to generate immunotoxins; the most favorable of which are DT389 and DT390 that possess different targeting moieties (**Figure 4B**) (45). The manipulation of the “B” domain has been shown to significantly augment the cytotoxicity of DT toward cancerous cells (47).

Moreover, DT recombinant derivatives with “B” domains with variable lengths have exhibited promising cytotoxic potentials. For example, DT with deletion of 97 amino acids (DT389) showed high antitumor activity, while the deletion of 191 amino acids reduced the toxin’s cytotoxicity by 1,000-fold compared to the native toxin (**Figure 4B**). Structural studies have shown that the “B” domain (i.e., the membrane-binding domain) is necessary to deliver the “A” domain to tumor cells (47).

Vallera et al. designed and synthesized a new bispecific DT-based immunotoxin (DT2219) against CD19- and CD22-positive cells. Three different immunotoxins were investigated *in vitro* for their anticancer activity against CD19+CD22+ Daudi or Raji cells. In general, DT2219 showed greater anticancer activity *in vitro* compared to monomeric and bivalent anti-CD19 and anti-CD22 immunotoxins. Further investigation showed that compared with parental anti-CD19 and anti-CD22 immunotoxins, DT2219 had higher binding affinity for leukemic cells (48). In another study, a DT-based immunotoxin carrying a truncated form of DT (consisting of the “A” and “T” domains) and a bivalent single-chain fused protein, Bic3 (anti-human CD3), was evaluated for its anticancer activity against CD3-ϵ-expressing human leukemic T cells. The results showed that the bivalent immunotoxin had higher binding affinity and lower toxicity than its monovalent form (49).

Recently, a DT-based anti-CC chemokine receptor 4 (CCR4) immunotoxin showed considerable antiproliferative activity against human CCR4+ tumor cells. Binding analysis revealed that the bivalent immunotoxin was more potent than its monovalent counterpart in recognizing human CCR4+ tumor cells and CCR4+ peripheral blood mononuclear cells (PBMCs). The highest binding affinity was related to the single-chain fold-back diabody isoform. In addition, the bivalent isoform displayed the most potent anticancer activity, which was 20-fold higher than that of the monovalent anti-CCR4 immunotoxin (50).

#### 3.1.3 Other bacterial toxin-based immunotoxins

Sarnovsky et al. (51) developed a new immunotoxin by combining the exotoxin of *Vibrio cholera* (CET40) and the scFv of human transferrin (HB21). The chimeric HB21-CET40 immunotoxin was reported to suppress the proliferation of cancer cell lines, including DLD-1, A549, KB3-1, 293TT, Raji, and HUT102, with the most potent activity being observed against the DLD-1 cell line. The new immunotoxin was screened for its cross-reaction with anti-PE antibodies *in vivo*, revealing ~50% similarity between the cholera exotoxin and PE. After the concomitant administration of the HB21-CET40 immunotoxin and anti-PE antibodies, the antibodies lost their HB21-CET40 neutralization capability (51).

Shiga toxin A (STXA) and *Campylobacter jejuni* cytolethal distending toxin B (Cj-CdtB) have also been used to develop immunotoxins (52–54). Goleij et al. synthesized two immunotoxins; PE38-anti-herceptin and recombinant PE-STXA-anti-herceptin. The recombinant immunotoxin was constructed by the coupling of the D-II domain of PE38 with the STXA of Shiga toxin (ST) *via* the recombinant DNA technology. In this structure, the D-II domain was responsible for the translocation of the cytolethal domain of STXA into the cytoplasm. These immunotoxins induced cell death in HER2-positive breast cancer cells (SKBR-3). Furthermore, PE38-STXA-anti-herceptin was a potent killer of SKBR-3 cells with 54% growth inhibition at the 100-µM concentration, showing anticancer activity close to that of PE38-anti-herceptin (i.e., 64% growth inhibition at the same concentration). None of the immunotoxins showed antiproliferative activity against the MCF-7 cell line (i.e., HER2-negative breast cancer cells), indicating their specific activity against HER2-positive cancer cells (53). Vafadar et al. (54) designed a new immunotoxin containing an anti-HER2 monoclonal antibody (trastuzumab) and Cj-CdtB. *In silico* findings showed that the new immunotoxin was soluble in aqueous media, had high stability, and showed selective targeting of HER-2 protein. However, further studies are needed to fully divulge the *in vitro* and *in vivo* biological activities of this immunotoxin (54).

Huang et al. (55) developed a Shiga-like toxin-A (3f7)-based immunotoxin against CD20-expressing cells, a hallmark of neoplastic B lymphocytes, which was shown to inhibit the growth of the cancerous cells partly *via* inducing apoptosis. The modified 3f7 was attached to CD20-specific scFv, which as a putative immunotoxin (MT-3724) triggered mitochondrial apoptotic pathways. Also, the MT-3724 immunotoxin displayed significant dose-dependent anticancer and antiproliferative activities against chronic myelogenous leukemia (CML) cell lines and in patient-derived xenograft (PDX) mouse models. *In vitro* experiments indicated that the MT-3724 immunotoxin significantly increased apoptosis in CML cell lines (IC_50_ values from 78 to 1,383 ng/ml). Furthermore, this immunotoxin showed remarkable anticancer activity against ibrutinib (a small-molecule drug that inhibits B-cell proliferation)-sensitive and ibrutinib-resistant cell lines with no significant change in the respective IC_50_ values, suggesting that the new immunotoxin could inhibit cell growth *via* different pathways from those of ibrutinib (55).

Mutter et al. (56) designed a novel generation of immunotoxins, called modular nanopore immunotoxins. In order to operate, these immunotoxins do not need cellular internalization, which is a prerequisite step for conventional immunotoxins. The nanopore-forming immunotoxin consisted of three distinct domains: a folate or nanobody [7d12 anti-epidermal growth factor receptor (EGFR) nanobody], a nanopore toxin-based moiety derived from *Salmonella typhi* [cytolysin A (ClyA)], and a protease domain (responsible for targeting cancer cells, reducing off-target toxicity against normal cells). The *in vitro* results showed that the folate-based nanopore immunotoxin was able to form pores on KB cells (human epithelial carcinoma cells) overexpressing folate receptors (FRs). The IC_50_ of ClyA was higher than that of ClyA-folate (13.5 vs. 5.45 nM). In addition, ClyA-folate had no toxicity against FR-negative cells. This finding suggested that the presence of the folate moiety was important for the formation of nanopores on FR^+^ cells. When the folate moiety was replaced with anti-EGFR nanobody, the anticancer activity of ClyA nanobody increased compared to low-cysteine ClyA (the IC_50_ values of 7.2 and 17.1 nM, respectively, against A431 epidermoid carcinoma cells overexpressing EGFR). On the other hand, the presence of EGF competitively blocked the specific anticancer activity of these immunotoxins. Furin-mediated cleavage, as the main advantage of these recombinant immunotoxins, was shown to abolish the off-target cytotoxicity of ClyA (56).

### 3.2 Ligand-derived chimeric anticancer toxins

Efforts are underway to find suitable alternatives for the antibody part of immunotoxins. For this purpose, a ligand, usually an immune ligand, is used as the targeting component. Some of these potential immune ligands have been described below.

#### 3.2.1 Interleukin-2

The promising results of clinical trials led the FDA to approve the DT389-Interleukin-2 (IL-2) immunotoxin (denileukin diftitox) for clinical use (**Table 1**) (57). Clinically applicable DT derivatives are developed by removing nonessential hydrophobic sequences (97 amino acids) of the “B” domain, which reduces their immunogenicity, boosts their cytotoxicity, and extends their half-life compared to DT486. Moreover, a DT389-derived chimeric toxin showed a longer lifetime and a higher specific response rate compared to the first-generation DT486-IL-2 chimeric toxin (13% vs. 37%) in patients with chemotherapy-refractory lymphomas (45, 57).

In another study, a chimeric IL-2 toxin showed selective toxicity against eukaryotic cell lines expressing high-affinity IL-2 receptors. Also, Bacha et al. (58) demonstrated that a chimeric IL-2 toxin specifically targeted IL-2 receptors on a variety of human transformed T-lymphotropic virus type (HTLV)-I-infected T lymphocytes and the murine interleukin-2-dependent CTLL-2 T cell line. Accordingly, the IL-2 toxin-mediated inhibition of protein synthesis in target T cells was reported to be blocked by IL-2R ligands (e.g., rIL-2 or anti-IL-2R mAb) (58).

Moreover, IL-2-diphtheria toxin-related fusion protein (IL-2-toxin) rapidly inhibited protein synthesis in IL-2R-expressing phytohemagglutinin-activated T cells, accompanied by transient induction of DNA synthesis. Seven hours after interaction with IL-2R^+^ phytohemagglutinin-activated T cells, IL-2-toxin-treated cells showed elevated mRNA levels of c-myc, interferon γ, and IL-2R. The results of Walz et al. (59) suggested that IL-2-toxin could affect IL-2 gene transcription/mRNA stabilization *de novo*, mediated by the IL-2R-binding domain and ADP-ribosyl transferase activity of the fused protein. Although the interaction of IL-2-toxin with IL-2R^+^ T cells initially increased the expression of c-myc (a transcription factor aberrantly expressed in over 70% of human cancers), interferon γ, IL-2R, and IL-2, these alterations were probably compromised by the inhibition of protein synthesis (59).

#### 3.2.2 Interleukin-3

Tagraxofusp (previously known as SL-401) is another FDA-approved DT-derived chimeric toxin used to treat blastic plasmacytoid dendritic cell neoplasms (BPDCNs) (**Table 1**). Tagraxofusp consists of IL-3 and DT388, and after intravenous administration, it binds to the alpha chain of IL-3 receptor, which is overexpressed on certain cancerous blood cells. Subsequently, DT388 is released into the cytoplasm of cancer cells where it mediates the ADP-ribosylation of EF-2, inhibiting protein synthesis and leading to cell death (60). Overall, regarding the potential cytotoxicity of DT389 and the overexpression of IL-3 on a wide range of cancerous cells, the efficacy of tagraxofusp has been evaluated in numerous clinical trials against hematologic neoplasms such as acute myeloid leukemia (AML), myelodysplastic syndrome (MDS), chronic myelomonocytic leukemia (CMML), BPDCN, and multiple myeloma (MM) (61).

#### 3.2.3 Interleukin-13

IL-13 is a glycosylated peptide showing limited but significant homology with the N- and C-terminal domains of IL-4, which are important for receptor binding. Normal blood cells express functional pleiotropic responses to IL-13 (62). Debinski et al. (63) generated a new recombinant CAT consisting of human IL-13 (hIL-13) and PE38QQR, a mutant form of PE. The cytotoxic action of hIL-13-PE38QQR requires its receptor-mediated internalization, which was blocked by an excess of hIL-13 but not of hIL-2. This process was shown to be hIL-13-specific, and excess hIL-4 was reported to block the cytotoxicity of hIL-13-toxin. Meanwhile, hIL-13 was noted to suppress the cytotoxicity of chimeric hIL4PE38QQR toxin (63).

In another study, IL-13-PE immunotoxin significantly and selectively decreased the viability of cancer cells expressing the cognate receptor and increased apoptotic/necrotic cell death in the NCI-H460 (human non-small cell lung carcinoma) cell line. The results demonstrated that IL-13-PE could be a therapeutic agent for IL-13Rα2-positive tumors. The cell-based delivery system for recombinant immunotoxins developed in the recent study can facilitate the clinical use of toxin therapy for treating various cancers (64).

#### 3.2.4 Programmed cell death protein-1

Mousavi et al. (65) designed a chimeric toxin consisting of mouse programmed cell death protein-1 (PD1) genetically fused to the “A” subunit of DT (DT386). The DNA construct was cloned and expressed in a bacterial system, then it was purified and identified by Western blotting. The chimeric toxin’s potency in eradicating tumors in C57BL/6 mice was evaluated. The chimeric toxin was injected into the tumors on eight occasions, which reduced the tumor volume by 67% compared to control animals [i.e., tumor-bearing mice treated with phosphate-buffered saline (PBS)], suggesting the therapeutic potential of the PD1-DT chimeric toxin for eradicating solid tumors (65).

#### 3.2.5 Human transferrin

Several recombinant DT derivatives have been developed by the deletion of variable lengths of the “B” domain. Greenfield et al. (66) constructed and characterized recombinant DT derivatives (CRM102, CRM103, and CRM107) carrying several point mutations in the “B” domain. Among these, CRM107 had more potent antiproliferative activity against Vero and Jurkat cells (66). In addition, the Tf-CRM107 recombinant toxin was shown to have anticancer effects against progressive or recurrent glioblastoma and anaplastic astrocytoma. The cytotoxic synthetic component of this chimeric toxin consisted of two main parts: human transferrin and CRM107 (a mutant form of DT). Point mutations in the cytolethal moiety of DT were shown to reduce the chimeric toxin’s nonspecific targeting. Although the results of phase I clinical trials have been promising in terms of safety, these CATs have not yet entered phase II and III trials (**Table 2**) (67).

#### 3.2.6 Transforming growth factor-beta

The superantigens of *Staphylococcus aureus*, including SE-A, B, and C, are under attention to be used for developing CATs, causing the superactivation of CD4+, CD8+, and gamma-delta T cells (68). In preclinical studies, superantigens have been tried to be targeted toward tumors *via* being conjugated with monoclonal antibodies and tumor-specific ligands (69, 70). In a study by Imani-Fooladi et al. (71), a genetically fused protein, transforming growth factor-alpha (TGFαL3)-staphylococcal enterotoxin type B (SEB), was designed as a novel antitumor candidate. This fused protein was constructed by conjugating the third loop of TGFαL3 with SEB.

The binding affinity of the transporter-associated antigen processing (TAP) of the TGFαL3-SEB fused protein was predicted by the TAPPred technique. In comparison with SEB, there was only one additional TAP-binding sequence in the TGFαL3-SEB fused protein. The epitopes of cytotoxic T lymphocytes (CTLs) restricted to 12 major histocompatibility complex (MHC) class I supertypes were predicted in the chimeric protein by the NetCTL 1.2 server using artificial neural networks (ANNs) (71).

## 4 Chimeric anticancer toxins with bacteria-derived targeting domains

### 4.1 Bacterial toxins

Considering the low remedial index of available cytotoxic drugs and the capacity of cancer cells to become resistant to these manufactured medications, it is essential to develop novel treatments for aggressive malignancies (7). Constructing chimeric bacterial toxins conjugated with cytotoxic agents *via* genetic engineering can help improve the anticancer properties of these compounds (72). In this regard, bacterial toxins may be combined with either immunomodulators or chemotherapeutics (7). The B-subunit of Shiga-like toxin (STXB) has been shown to present anticancer effects *via* inducing apoptosis and inhibiting cell cycle progression (72). Other studies have also shown that ST, *via* binding to specific surface receptors, can be an effective transporter for delivering toxic proteins to cells. The conjugation of a molecule to ST can change the toxin’s properties such as stability and immunogenicity and enhance its toxic effects. In addition, ST is relatively small, and pairing it with larger molecules can improve its binding efficacy to globotriaosylceramide 3 (GB3) (8, 72), a functional alternative receptor for ST. The overexpression of GB3 on the surface of malignant cells has been reported in primary breast cancer, ovarian cancer, gastric adenocarcinoma, and acute nonlymphocytic leukemia (72). In a study, the anticancer properties of a chimeric protein consisting of STXB and DT were investigated (73), reporting that the effects of DT389-STXB chimeric protein were strongly associated with the expression level of GB3 (i.e., higher toxicity against the T47D and 4T1 cell lines overexpressing the GB3 receptor) (73).

As a nontoxic toxin with low immunogenicity, STXB specifically binds globotriaosyl-ceramide (Gb3/CD77), which is highly expressed on some human tumors such as pancreatic, colon, and breast and acts as a functional receptor for ST. So, this toxin can be applied to target Gb3-positive human tumors. In a study, Mohseni Moghadam et al. constructed the DT390-STXB chimeric protein *via* fusing the DT390 fragment of DT538 (i.e., native diphtheria toxin) to STXB and then evaluated its antitumor effects. The results demonstrated that the codon adaptation index (CAI) increased from 0.6 (for the wild-type gene) to 0.9 (for the *dt390-stxB* chimeric gene) (74).

It has been demonstrated that *Vibrio vulnificus* produces a RAS/RAP1-specific endopeptidase (RRSP) that disrupts the RAS signaling pathway. In a recent study, Vidimar et al. (75) designed and synthesized a chimeric toxin consisting of DTa and RRSP, which was shown to have high affinity for RRSP and kill tumor cells more effectively. They further produced the RRSP-CPD-DTa-DTB chimeric toxin by adding an autoprocessing cysteine protease domain (CPD) through the *V. vulnificus* MARTX to increase RAS cleavage and protein release, which enhanced its toxicity against TNBC cells (75). In another study, Kakutani et al. (76) investigated the anticancer effects of a recombinant protein consisting of the C-terminal fragment of *Clostridium perfringens* enterotoxin (C-CPE) and diphtheria toxin domain A (DTA). Cancer studies showed that CL-1, -2, and -4 are frequently expressed in L cells, and that DTA–C-CPE had remarkable cytotoxicity against CL-4+ L cells (76).

It has been shown that C-CPE can bind claudin-4 (CL-4) (77), a tight junction protein highly expressed in some cancers, such as pancreatic, breast, prostate, and ovarian (78). The anticancer activity of CPE has been shown against claudin-expressing breast and pancreatic cell lines (76). CL-4 bound to DTA–C-CPE can be internalized by endocytosis, followed by the release of DTA from endosomes into the cytosol (64). The potential of these bacteria-derived CATs for targeting cancer cells is of great research interest today.

### 4.2 Antimicrobial peptide-derived chimeric anticancer toxins

Antimicrobial peptides (AMPs) are small, cationic, hydrophobic, and amphipathic host defense molecules that play a key role in the functioning of cytoprotection systems against pathogenic viruses, bacteria, and fungi. Due to their cationic and amphipathic nature causing electrostatic interactions, AMPs form an important class of anticancer and antibacterial agents that can penetrate into cancer cells and through the cell walls of bacteria. Several studies have evaluated the anticancer activities of AMPs (79, 80). Membrane modifications are commonly seen in tumor cells; for example, these cells generally have more negatively charged membranes compared to normal cells, facilitating their binding to AMPs (81). Natural and engineered AMPs have been studied as sources for developing novel anticancer drugs with a broad range of biological activities (82).

Shafiee et al. (83) designed and synthesized new CATs by combining truncated DT (i.e., DT386, domains A and T) with a buforin II-derived antimicrobial peptide (BR2) (**Figure 3**). In this structure, BR2 facilitated the penetration of CAT into cancer cells, enhancing the cytoplasmic accumulation of DT389. Compared to DT386, DT386-BR2 exhibited more potent *in vitro* antitumor activity against K-562 cells (a human-derived CML cell line, IC_50_ = 0.8 vs. 2.05 μg/ml). Additionally, DT386-BR2 displayed time- and dose-dependent proapoptotic activity against cancerous cells (83).

Azurin, a copper-containing redox protein (cupredoxin), is produced by *P. aeruginosa*. A small truncated form of azurin (Leu50–Asp77, also known as p28, responsible for transferring azurin into host cells) was shown to induce p53-mediated apoptosis in cancer cells (84). Likewise, p28 has been successfully employed to deliver chemotherapeutics such as doxorubicin, dacarbazine, temozolomide, paclitaxel, and docetaxel to human cancer cells expressing either wild-type or mutated p53, improving the therapeutic efficacy of these drugs and reducing the doses administered (84). Recently, Soleimani et al. (85) designed and synthesized a CAT containing two AMPs, p28 and NRC (a cationic antimicrobial peptide with anticancer activity). The *in vitro* results showed that the p28-NRC fused peptide had remarkable antiproliferative activity against the MCF7 and MDA-MB-23 breast cancer cell lines in a time- and dose-dependent manner (minimal 48-h IC_50_ values of 1.88 and 1.89 µM, respectively) (85). Furthermore, many bacteria-derived AMPs have presented anticancer properties *in vitro* and *in vivo*, which can be used to generate novel chimeric toxins to combat cancer (86).

### 4.3 Affibody

Affibody is a small cell-penetrating molecule derived from the Z domain of *Staphylococcus aureus* protein A (SP-A) (87). One of the major challenges of immunotoxin-based cancer therapy is the relatively large sizes of the antibodies used. However, affibodies are 20 times smaller than antibodies and four times smaller than scFvs. The nano-dimension, high binding affinity, and specificity of affibodies render them suitable alternatives to scFv-based immunotoxins (88). Zielinsk et al. (89) constructed an affibody-based chimeric toxin, named affitoxin, which consisted of a HER2-specific affibody and the PE38KDEL toxin. Their results showed that the IC_50_ of the CAT for HER2-positive MCF-7 cells was 20 times lower than that for HER2-negative MDA-MB468 cells (IC_50_ = 2.56 vs. 62 pM). These results suggested that affibodies could be used as potential targeting moieties to develop novel anticancer chimeric toxins.

## 5 Manufacturing processes and challenges

There are currently three bacteria-derived immunotoxins approved for clinical use in the United States (8–10). Since it is uncertain whether or not health insurers will cover these medications, the high cost of novel antibody–drug conjugates (ADCs) may be a barrier to their widespread use (90). Immunotoxins consist of a toxin moiety and a targeting moiety (e.g., mABs or their fragments), and they are more costly and complicated to be manufactured than standard mABs. Immunotoxins are produced by combining three components: mAb, microbial/recombinant toxin, and a linker (91), each of which must be prepared and purified separately before being conjugated. Three platforms are available: a mammalian cell platform for mAB production in CHO cells, an *E. coli* platform for toxin expression, and a chemical synthesis platform for linker assembly. Immunotoxin production using mAB fragments is more straightforward than ADC production because no chemical coupling or intermediates are required (90).

### 5.1 Expression hosts

Because toxins are cytotoxic to eukaryotic cells, mammalian host cells are susceptible to toxin components. Therefore, either the expression host needs to be resistant against the toxin and its derivatives or toxin cargoes should be able to spatially divert the toxin from its molecular target, which can increase product yield (92). In one example, a toxin-resistant CHO cell line was developed, but product yield was significantly reduced during cell engineering (0.004 g^-1^ for diphtheria-based immunotoxins and 5 g^-1^ for mABs) (93). Although posttranscriptional modifications, such as N-linked glycosylation, do not occur in prokaryotic hosts, *E. coli* is a common host used for immunotoxin production (94); however, protein misfolding and the subsequent costly processes of refolding are major drawbacks (95). Protein denaturation and refolding impede the large-scale production of immunotoxins in prokaryotic hosts, encouraging the use of alternative hosts such as insects, yeasts, and plants (96). Compared with mammalian cells, plant cells offer simplified and relatively cheap expression systems (97). It is possible to produce mAbs and their derivatives (such as scFvs, nanobodies, and multibodies), as well as bacteria-based immunotoxins, using plant expression systems such as intact transgenic plants, plant cell cultures, and *Agrobacterium*-mediated expression in wild tobacco plants (96, 98, 99).

### 5.2 Designing and modification of bacterial toxins

The adverse effects of immunotoxins necessitate their chemical inactivation, which is among the first modification steps during the production process (96). For bacterial toxins, several molecular techniques have been developed to reduce their undesirable effects, including the removal of nonspecific binding domains and immunogenic epitopes (100). As another modification, the fusion of toxins with translocation domains, such as PE domain II, enables them to cross biological membranes (101).

### 5.3 Linker

To design efficient immunotoxins, it is important to use rigid, flexible, and cleavable linkers (102). The use of flexible linkers facilitates protein folding by allowing the peptide backbone to twist and bend, which can be achieved by using small amino acids such as glycine (103). There are two types of rigid linkers: 1) alpha-helical linkers stabilized by electrostatic interactions between the side chains of glutamic acid and lysine and 2) non-helical linkers stabilized by proline (102). On the other hand, cleavable linkers can form intramolecular disulfide bridges and facilitate the release of intracellular toxins by the action of proteases. A cleavable linker was successfully constructed using a peptide containing the amino acid sequence of the translocation domain of diphtheria toxin (AGNRVRRSVG) (104). An efficient linker should be able to transport the cargo into the target cell and subsequently mediate its release *via* proteolytic cleavage (102). Overall, for designing a biologically active bacteria-derived immunotoxin, multiple components should be optimally juxtaposed, including the targeted antigen, expression platform, targeting antibody, toxin, and linker.

## 6 Conclusions

Bacterial toxins, alone or in combination with conventional chemotherapies, can increase the effectiveness of cancer treatments. Several bacterial toxins have been studied as anticancer agents, exhibiting antiproliferative and proapoptotic properties. Novel chimeric toxins are evolving to obviate the limitations of available cancer medications, such as high immunogenicity, off-target toxicity, and drug resistance. Structural modifications in primary bacterial toxins, such as removing or replacing toxin domains or introducing mutations in them, can improve their stability and cytotoxicity against cancerous cells while reducing their side effects. In preclinical studies, efforts are underway to produce recombinant toxins conjugated with antibodies, specific ligands, and immune effectors. So far, three types of immunotoxins (denileukin diftitox, Tagraxofusp-erzs, and moxetumomab pasudotox) have acquired FDA approval for clinical use. However, despite being promising in clinical trials and even receiving FDA approval, other available immunotoxins require further *in vitro* and *in vivo* investigations to verify their effectiveness and safety (**Tables 1**, **2**).

## Author contributions

MH designed the study, manuscript initiation, organization, revision and submission. BN, SK, HF, MA and FN wrote the manuscript. MH and PB designed and illustrated the Figures and Table. All authors contributed to the article and approved the submitted version.

## Conflict of interest

The authors declare that the research was conducted in the absence of any commercial or financial relationships that could be construed as a potential conflict of interest.

## Publisher’s note

All claims expressed in this article are solely those of the authors and do not necessarily represent those of their affiliated organizations, or those of the publisher, the editors and the reviewers. Any product that may be evaluated in this article, or claim that may be made by its manufacturer, is not guaranteed or endorsed by the publisher.

## Glossary

**Table:**

AML	acute myeloid leukemia
ADPR	adenosine diphosphate ribosyl
ADCs	antibody–drug conjugates
AMPs	antimicrobial peptides
ANNs	artificial neural networks
BPDCNs	blastic plasmacytoid dendritic cell neoplasms
BR2	buforin II
CPE	Clostridium perfringens enterotoxin
CCR4	CC chemokine receptor 4
CATs	chimeric anticancer toxins
CML	chronic myelogenous leukemi a
CMML	chronic myelomonocytic leukemia
CL-4	claudin-4
BoNT	Clostridium botulinum neurotoxin
CAI	Codon Adaptation Index copper-containing redox protein
C-CPE	C-terminal fragment of Clostridium perfringens enterotoxin cupredoxin
ClyA	cytolysin A
CNF	cytotoxic necrotizing factor
CTL	cytotoxic T lymphocyte
PD1	programmed cell death protein-1
DT	diphtheria toxin
DTA	diphtheria toxin domain A
EF-2	elongation factor 2
EGFR	epidermal growth factor receptor
FR	folate receptor
GB3	globotriaosylceramide 3
HER2	human epidermal growth factor receptor 2
hIL-13	human IL-13
HTLV	human T-lymphotropic virus type
MTD	maximum tolerated dose
MSLN	mesothelin
MM	multiple myeloma
MDS	myelodysplastic syndrome
PBMCs	peripheral blood mononuclear cells
PBS	phosphate-buffered saline
PARP	poly ADP-ribose polymerase
STXA	Shiga toxin A
SEB	Staphylococcal enterotoxin type B
SP-A	Staphylococcus aureus protein A
TGF-a	transforming growth factor alpha
TAP	transporter-associated antigen processing
FDA	US Food and Drug Administration.
